# Manejo estomatológico del paciente con hepatopatías: una revisión de la literatura

**DOI:** 10.21142/2523-2754-1102-2023-153

**Published:** 2023-06-29

**Authors:** Javiera Cancino, Sebastián Lazo, Diego Fonseca

**Affiliations:** 1 Facultad de Odontología, Universidad Finis Terrae. Santiago, Chile. jpcancino@miuandes.cl Universidad Finis Terrae Facultad de Odontología Universidad Finis Terrae Santiago Chile; 2 Facultad de Odontología, Universidad del Desarrollo. Santiago, Chile. seba.lazo.r@gmail.com Universidad del Desarrollo Facultad de Odontología Universidad del Desarrollo Santiago Chile seba.lazo.r@gmail.com; 3 Facultad de Odontología, Universidad de los Andes. Santiago, Chile. d.fonsecaescobar@gmail.com Facultad de Odontología Universidad de los Andes Santiago Chile d.fonsecaescobar@gmail.com

**Keywords:** cirugía bucal, hepatopatías, cirrosis hepática, hemorragia bucal, oral surgery, liver disease, hepatic cirrhosis, oral bleeding

## Abstract

**Introducción:**

El hígado es el órgano encargado del metabolismo de nutrientes y algunos fármacos, así como de producir factores de coagulación. Según la Organización Mundial de la Salud, cada año se registran aproximadamente 23 millones de personas con patologías hepáticas a nivel mundial; por tanto, es habitual que el odontólogo se encuentre con estos pacientes en las consultas diarias. El objetivo de esta revisión es establecer el manejo estomatológico de los pacientes que presentan hepatopatías.

**Materiales y métodos:**

Se realizó una búsqueda de artículos indexados a las bases de datos de PubMed y EBSCO utilizando una combinación de palabras clave y los términos booleanos “oral surgery” AND “hepatitis” AND “dental management”.

**Resultados:**

Los pacientes con hepatopatías presentan importantes características para el odontólogo, las cuales deben ser reconocidas para que los procedimientos puedan ser realizados con el menor riesgo de complicaciones intra y posoperatorias. Un paciente con mal control de su patología de base se encuentra más propenso a infecciones y hemorragias, lo que implica un alto riesgo de morbilidad.

**Conclusiones:**

Se debe valorar la atención odontológica del paciente con hepatopatías según el motivo de consulta, el control de la enfermedad, la complejidad del procedimiento a realizar y las medidas hemostáticas tanto intra como posoperatorias. Se deben considerar todas las medidas hemostáticas necesarias y considerar ajuste de dosis en el uso de AINE.

## Abstract (en)

**Introduction:**

The liver is the organ responsible for the metabolism of nutrients, some drugs, and the production of coagulation factors. According to the World Health Organization, approximately 23 million people worldwide are diagnosed with liver disease each year. As a result, it is common for dentists to encounter these patients on a daily basis in their practice. The objective of this review is to establish the dental management of patients with liver disease.

**Material and methods:**

A manual literature search was conducted using the indexed articles in PUBMED and EBSCO databases using the keywords “oral surgery,” AND “liver disease,” AND “hepatic cirrhosis,” AND “dental management”.

**Results:**

Patients with liver disease present important characteristics for the dentist, which must be recognized in order to perform procedures with the lowest risk of intraoperative and postoperative complications. A patient with poorly controlled underlying liver disease is more prone to infections and bleeding, which implies a high risk of morbidity.

**Conclusions:**

Dental care for patients with liver disease should be assessed according to the reason for consultation, control of the disease, the complexity of the procedure to be performed, and both intraoperative and postoperative hemostatic measures. All necessary hemostatic measures should be considered and dose adjustments should be considered in the use of NSAIDs.

## INTRODUCCIÓN

El hígado desempeña un papel fundamental en diversos procesos fisiológicos, desde el metabolismo de grasas hasta la homeostasis ^1^. El paciente con patología hepática presenta una serie de particularidades que deben tenerse en cuenta antes de cualquier intervención. El odontólogo tiene el deber de evaluar la atención y considerar cualquier alteración en la hemostasia, el metabolismo de drogas/fármacos y las manifestaciones orales en estos pacientes, con el fin de minimizar cualquier complicación durante y después de todo tipo de intervención ^2^.

El presente artículo tuvo como objetivo establecer el manejo estomatológico del paciente con hepatopatías mediante una revisión narrativa de la literatura.

## MATERIALES Y MÉTODOS

Se realizó una búsqueda manual de revisión bibliográfica en las bases de datos de PubMed y EBSCO por dos investigadores (JC; SL) de manera independiente, utilizando una combinación de palabras clave y los términos booleanos “oral surgery“ AND “hepatitis” AND “dental management”. En cuanto a los criterios de inclusión, se consideraron revisiones bibliográficas, estudios observacionales, ensayos clínicos, guías clínicas, revisiones sistemáticas y metaanálisis publicados entre noviembre de 2013 y 2023, en inglés o español. Se excluyeron estudios en animales y cartas al editor. Finalmente, 43 artículos fueron incluidos en esta revisión. 

## RESULTADOS Y DISCUSIÓN

### Consideraciones sobre la anatomía

El hígado es la glándula de mayor tamaño en el cuerpo y, después de la piel, es el órgano individual más grande. Su unidad funcional son los hepatocitos. Se encuentra ubicado en el cuadrante superior derecho del abdomen, protegido por la caja torácica y el diafragma. A excepción de las grasas, todos los nutrientes absorbidos por el tracto gastrointestinal convergen inicialmente en el hígado, a través del sistema venoso portal ^3^. Entre sus funciones se encuentran el procesamiento de sustancias absorbidas; la síntesis y secreción de ácidos biliares; la producción y expresión de bilirrubina; el metabolismo de nutrientes como carbohidratos, proteínas y lípidos; y la desintoxicación y excreción de productos de desecho ^4^.

### Consideraciones sobre la función

#### Producción de bilis

La bilis es un fluido producido por los hepatocitos, compuesto principalmente por agua, electrolitos, sales y ácidos biliares, colesterol, pigmento biliar, bilirrubina y fosfolípidos, entre otros elementos. Esta sustancia ayuda a excretar materiales que no se eliminan a través del riñón y facilita la absorción y digestión de lípidos mediante la secreción de sales biliares y ácidos ^5^.

#### Producción de bilirrubina

La hemólisis tiene lugar en múltiples zonas del organismo, incluidos el hígado, el esplenio y la médula ósea. En un principio, el grupo heme es fraccionado a biliverdina peridante, la heme oxigenasa, que luego vuelve a fraccionarlo en bilirrubina mediante biliverdina reductasa ^6^. En su forma no conjugada, la bilirrubina es insoluble en agua y es transportada al hígado unido a albúmina, donde es conjugada con ácido glucurónico, para luego ser secretada en los canalículos biliares ^7^. Una vez secretada junto a la bilis en el tracto intestinal, es desconjugada por bacterias intestinales a urobilinógenos. Estos son posteriormente oxidados y reabsorbidos en la circulación enterohepática y secretados en la bilis. El color amarillo característico de la orina se debe a los compuestos oxidados de urobilinógenos ^8^.

#### Metabolismo de los nutrientes

El hígado se encarga de mantener el equilibrio de proteínas y sus productos de desecho, como es el caso de la formación de urea. Sintetiza cerca del 90% aproximadamente de las proteínas plasmáticas, como albúmina, alfafetoproteína, fibrinógeno, protrombina, colágeno y la producción de numerosas enzimas que participan en la formación de carbohidratos, grasas y alcohol, como transaminasa glutámico-pirúvica (TGP), alanina aminotransferasa (ALT), fosfatasa alcalina (FA), alcohol deshidrogenasa y gamma-glutamil transpeptidasa (GGT) ^9^.

Participa, además, en el almacenamiento de glucógenos, la conversión de galactosa y fructosa en glucosa, y la producción de glucosa a partir de precursores no glucosídicos ^8^.

En cuanto al metabolismo de los lípidos, el hígado lidera la beta-oxidación de ácidos grasos y la formación de ácido acetoacético. Asimismo, participa en la formación de gran parte de las lipoproteínas necesarias para el transporte de colesterol; la síntesis de colesterol, triglicéridos y fosfolípidos; y la conversión de grandes cantidades de carbohidratos y proteínas en grasa ^3^.

#### Metabolismo de fármacos

En este órgano, múltiples drogas pueden ser metabolizadas, lo que tiene como resultado la producción de compuestos solubles en agua que pueden ser excretados en la bilis ^10^. El hígado es capaz de transformar xenobióticos, principalmente convirtiéndolos de una forma lipofílica a una hidrofílica mediante dos reacciones: fase I y fase II. La fase I es mediada por el citocromo P450, que incluye reacciones de oxidación, reducción e hidrólisis. La fase II, por otro lado, conjuga los metabolitos creados en la fase I para secretarlos en la bilis o la sangre ^5^^,^^10^.

### Consideraciones sobre la hepatitis

La hepatitis se define como la inflamación del hígado, la cual puede tener una variedad de causas, como el consumo excesivo de alcohol, la autoinmunidad, las drogas o las toxinas. Sin embargo, la causa más frecuente de hepatitis son las infecciones virales, en cuyo caso se conoce como hepatitis viral ^1^.

#### Hepatitis alcohólica

El consumo excesivo de alcohol causa enfermedad alcohólica del hígado y, en estadios más avanzados, hepatitis alcohólica. Cuando se considera al alcohol como una causa primaria y cofactor, se estima que aproximadamente el 30-50% de las muertes asociadas a cirrosis en todo el mundo están relacionadas con ello ^1^. La estimación de la hepatitis alcohólica no es sencilla, ya que depende del reporte de consumo de alcohol entregado por el paciente. Se espera que la prevalencia de esta enfermedad aumente debido al aumento global en el consumo de alcohol ^11^. Esta condición inflamatoria difusa del hígado se caracteriza por cambios celulares que pueden ser irreversibles ^8^. La evidencia actual sugiere que el daño producido en la hepatitis alcohólica es la culminación de una serie de interacciones entre el metabolismo del etanol, la inflamación y la inmunidad innata. Estas vías metabólicas generan especies reactivas de oxígeno que son inductores potentes de la peroxidación de lípidos, lo que provoca la muerte de hepatocitos por apoptosis o necrosis ^12^.

#### Hepatitis autoinmune

Es una enfermedad hepática inflamatoria inmunomediada de curso clínico no autolimitado, que puede afectar a todas las edades, ambos sexos y todas las etnias. En la mayoría de los pacientes afectados, se requieren agentes inmunosupresores. Esta enfermedad no tiene un signo patognomónico y el diagnóstico requiere la presencia de características clínicas y exámenes de laboratorio ^5^. El concepto de la inmunopatogénesis de la hepatitis autoinmune se basa en las células T CD4 y CD8 autorreactivas, cuya aparición se induce después de la ruptura de la autotolerancia por desencadenantes ambientales ^13^.

#### Hepatitis viral

Las infecciones crónicas por hepatitis viral conducen a graves problemas de salud, incluyendo hepatitis crónica, cirrosis, insuficiencia hepática e, incluso, carcinoma hepatocelular. Las hepatitis virales más comunes son la hepatitis B y la hepatitis C ^1^.

Se estima que aproximadamente 257 millones de personas viven actualmente con hepatitis B. Sin el tratamiento apropiado, aproximadamente el 25% morirá a causa de una falla hepática o carcinoma hepatocelular. Además, se estima que solo el 11% de los infectados son conscientes de su enfermedad y apenas el 17% de ellos recibe tratamiento ^14^. Entre las vías de transmisión se encuentran la vertical, sexual, nosocomial y el uso de drogas intravenosas ^14^^,^^15^. La patogénesis de la enfermedad hepática es principalmente inmunomediada y, en algunas circunstancias, puede causar citotoxicidad directa al hígado ^15^.

En cuanto a la hepatitis C, su ciclo de replicación depende en gran medida de la vía del metabolismo de los lípidos en los hepatocitos y altera considerablemente la homeostasis lipídica del huésped, lo que aumenta el riesgo cardiovascular ^15^. A nivel global, 71 millones de personas están infectadas con el virus y el 51% de ellas se concentran en países como India, China, Egipto, Rusia, Estados Unidos y Pakistán. La principal vía de contagio es la nosocomial y, en menor grado, la vertical y sexual ^16^.

### Exámenes de laboratorio

A menudo, es importante obtener un hemograma completo, recuento de plaquetas y parámetros de coagulación, como la relación internacional normalizada (INR), lo más cerca posible del momento del procedimiento. El INR es una de varias pruebas de laboratorio comunes que se utilizan para evaluar la coagulación. Históricamente, el INR se ha empleado para controlar a los pacientes con warfarina, pero también permite evaluar el estado de coagulación de los pacientes con enfermedad hepática crónica ^17^^,^^18^.

Entre los denominados test de función hepática (TFH) se incluyen varios parámetros bioquímicos como aspartato aminotransferasa (AST), alanina aminotransferasa (ALT), gamma-glutamil transpeptidasa (GGT), fosfatasa alcalina (FA), bilirrubina, albúmina y actividad de protrombina. Sin embargo, dicha terminología no es del todo exacta ya que, de todos ellos, solo los tres últimos miden la capacidad funcional del hígado, mientras que los demás son potenciales indicadores de daño hepático ^17^.

Los parámetros incluidos en dichas pruebas no son órgano-específicos y pueden reflejar tanto patología hepática como en otros niveles. Así, podemos encontrar hiperbilirrubinemia aislada en casos de hemólisis, elevaciones de la fosfatasa alcalina sin otros parámetros hepáticos alterados en casos de patología ósea o elevaciones de la ALT tras ejercicio físico, patología muscular o hipotiroidismo ^9^ (Tabla 1).

**Tabla 1:** Estudios bioquímicos hepáticos habituales en los trastornos hepáticos frecuentes

Enfermedad	AST	ALT	FA	Bilirrubina	Otras características
Hepatitis viral aguda	+++	+++	Normal o +	Normal o +	Exposición anterior, fatiga, náuseas
Hepatitis viral crónica	+	++	Normal o +	+ si es avanzada	Exposición anterior a sangre infectada o líquidos corporales
Hepatitis alcohólica	++	Normal o +	+	Normal o +++	Consumo excesivo de alcohol
Hepatitis autoinmune aguda	+++	+++	Normal o +	Normal o ++	Autoanticuerpos
Hepatitis autoinmune crónica	+	++	Normal o +	Normal	Autoanticuerpos

+, Aumentado

De todo esto se deduce que una adecuada interpretación de los resultados de cualquier prueba de función hepática debe ir siempre precedida por una rigurosa historia clínica en la que se recojan los síntomas y signos que presenta el paciente, con especial hincapié en potenciales factores de riesgo sexual o parenteral (transfusión, consumo de drogas por vía parenteral, etc.), existencia de enfermedades concomitantes y consumo de alcohol, fármacos o productos de herboristería ^19^^,^^20^.

### Manifestaciones orales del paciente con hepatitis

Las enfermedades hepáticas también tienen repercusión en la salud oral de quienes las padecen. Muchas de estas manifestaciones pueden explicarse por los factores de riesgo asociados, como el tabaco, la diabetes y el consumo de alcohol ^21^. En la cavidad oral se puede apreciar el compromiso de los tejidos duros (dientes), las glándulas salivales y las manifestaciones mucocutáneas ^22^.

#### Manifestaciones en los tejidos duros

El reflujo gastroesofágico es habitual en pacientes con daño hepático crónico. Esta regurgitación ácida puede causar erosión del esmalte dental si el pH cae por debajo de 5,5. Dicha situación puede agravarse si, además, existe polifarmacia y consumo de bebidas ácidas ^23^.

#### Manifestaciones mucocutáneas

Como se sabe, en pacientes con una función hepática alterada, la homeostasis también puede verse afectada, lo que en la cavidad oral se manifiesta como petequias o sangrado gingival excesivo en traumatismos menores ^24^.

Otra patología prevalente es la aparición de liquen plano, una condición inflamatoria común que puede afectar la piel y las mucosas, incluida la oral. Existe una fuerte conexión entre la infección por hepatitis C y la aparición de liquen plano. También se han reportado lesiones liquenoides y leucoplasia en las enfermedades hepáticas no virales. Una biopsia temprana es fundamental, ya que un bajo porcentaje de estas lesiones pueden progresar con un carácter maligno ^21^.

#### Glándulas salivales

En cuanto al compromiso de las glándulas salivales, se puede observar xerostomía ^25^. La xerostomía se refiere a la sensación subjetiva de boca seca. Se sabe que deteriora la calidad de vida en los pacientes que la padecen. La combinación de la patología hepática y los medicamentos indicados puede afectar el flujo salival. Además, incrementa la vulnerabilidad del paciente a presentar caries y trastornos en el tejido blando, lo que en combinación con una higiene oral deficiente puede facilitar el desarrollo de periodontitis y candidiasis ^21^^,^^22^.

#### Complicaciones en pacientes con hepatopatías

La enfermedad puede existir durante períodos prolongados de manera asintomática. Ocasionalmente, puede entrar en remisión clínica, pero con una continua necrosis hepatocelular o una progresión a cirrosis. Eventualmente, la mayoría de los pacientes desarrollan cirrosis ^26^.

Si los pacientes con la enfermedad clínica e histológicamente grave no son tratados adecuadamente, la tasa de mortalidad puede ser alta. La mortalidad es mayor durante los dos primeros años, cuando la enfermedad es más activa y cuando la enfermedad tiene un inicio repentino y severo. Durante los primeros años, la muerte del paciente generalmente se debe a insuficiencia y coma hepático. En etapas posteriores de la enfermedad, la muerte es comúnmente resultado de complicaciones por cirrosis, hemorragia varicosa, encefalopatía hepática o infecciones recurrentes ^27^.

Los pacientes con enfermedad hepática crónica pueden presentar cirrosis. La cirrosis no complicada se denomina “cirrosis compensada” y puede ser asintomática o cursar con síntomas inespecíficos. La cirrosis descompensada puede incluir complicaciones tales como ascitis, ictericia, carcinoma hepatocelular, síndrome hepatorrenal, hemorragia por rotura de várices esofágicas, peritonitis bacteriana espontánea y encefalopatía hepática ^28^^,^^29^.

La enfermedad hepática crónica es una de las principales causas de morbilidad y mortalidad ^30^. La esperanza de vida en estos pacientes ha incrementado y por ello el número de intervenciones quirúrgicas a las que estos pacientes se someten. Los cambios fisiopatológicos y hemodinámicos en la cirrosis hacen que estos pacientes sean más susceptibles a la hipotensión e hipoxia durante la cirugía. Además, tienen mayor riesgo de daño hepático inducido por fármacos, disfunción renal y descompensación hepática posoperatoria. Por ello, se debe evaluar detalladamente el riesgo preoperatorio en estos pacientes, especialmente por una hipertensión portal significativa y cirrosis ^30^^,^^31^.

### Manejo odontológico

#### Prevención de contagio y manejo en hepatopatías virales

Las infecciones en la consulta dental pueden ser transmitidas mediante una serie de vías, incluyendo el contacto con sangre, fluidos orales y otras secreciones. El VHB es el principal agente causal de enfermedad hepática crónica y aguda, cirrosis y carcinoma hepatocelular. Entre las exposiciones que pueden poner al dentista en riesgo de contagio se encuentran la injuria percutánea (pinchazo o corte con un objeto filoso), el contacto con sangre potencialmente infectada, tejidos no indemnes y la mucosa ocular, nasal y oral ^32^.

Para disminuir la transmisión de hepatitis viral en la práctica dental, se recomienda que los trabajadores reciban inmunización contra el virus de la hepatitis y utilicen equipos de protección individual como guantes, gorras, máscaras, etc. ^24^.

En el caso de los pacientes con un cuadro activo de hepatitis viral, se debe retrasar la atención electiva. En el caso de una urgencia, esta debe realizarse solamente en un box operatorio aislado con estricta adherencia a las precauciones estándar ^33^.

#### Valoración del riesgo hemorrágico

La tendencia al sangrado es característica en las enfermedades hepáticas avanzadas, lo cual se atribuye a un déficit de factores de coagulación, especialmente del grupo de protrombina (factores II, VII, IX y X), todos ellos dependientes de la vitamina K como precursor para su producción, cuyo almacenamiento y conversión en el hígado se ven disminuidos ^8^.

El INR proporciona poca información sobre el estado de la hemostasia en pacientes con hepatopatías. Aun así, sigue siendo ampliamente utilizado antes de los procedimientos quirúrgicos. Existen estudios que afirman su falta de utilidad para predecir con exactitud el riesgo de sangrado en enfermedades del hígado en pacientes sometidos a una serie de procedimientos médicos, incluyendo las extracciones dentales ^34^.

En pacientes con cirrosis hepática, es habitual presentar trombocitopenia (recuento plaquetario menor a 150 000 uL), lo cual ocurre en aproximadamente el 68% de los pacientes con un cuadro compensado ^35^. Aun así, a pesar de tener un recuento plaquetario bajo y un INR elevado, con un estado de coagulación y agregación plaquetaria alterada, se ha reportado que la prevalencia de eventos hemorrágicos en pacientes con cirrosis es del 0 al 8,9% ^2^.

Cualquier intervención odontológica debe ser valorada previamente para considerar las medidas hemostáticas que se utilizarán (Tabla 2).

**Tabla 2:** Valoración de riesgo de sangrado en procedimientos dentales según el Scottish Dental Clinical Effectiveness Programme

Bajo riesgo de sangrado	Alto riesgo de sangrado
Extracciones simples (1-3 dientes restringiendo el tamaño de la herida)	Extracciones complejas o adyacentes que causen una herida de mayor calibre
Incisión y drenaje	Más de 3 extracciones al mismo tiempo
Sondaje periodontal profundo	Colgajos
Desbridamiento de la superficie radicular	Biopsias
Restauraciones directas	Recontorneado gingival
Restauraciones indirectas con margen supragingival	

#### Necesidad de profilaxis antibiótica

En general, los pacientes con hepatopatías no requieren profilaxis antibiótica a menos que se encuentren en un cuadro de inmunosupresión avanzado, ya que pueden estar en riesgo de complicaciones como bacteriemia e infecciones en sitios distantes posteriores a la intervención dental. En general, se recomienda seguir las recomendaciones de la American Heart Association para prevenir la endocarditis bacteriana, según el riesgo de cada paciente ^36^.

#### Hemostasia

Las medidas de hemostasia dependen del grado de complejidad de la intervención a realizar. Se ha reportado que la prevalencia de eventos hemorrágicos en pacientes con cirrosis es del 0% al 8,9%, a pesar de poseer un recuento plaquetario bajo y un INR elevado ^2^.

Algunas medidas hemostáticas son suturas, rellenos alveolares y agentes antifibrinolíticos para el control del sangrado, siendo estos últimos los principalmente utilizados como medida de control de sangrado postoperatorio ^37^.

En cuanto a los agentes antifibrinolíticos descritos, destaca el ácido tranexámico (ATX), cuya presentación farmacológica (enjuague o inyectable) depende, en gran medida, de la disponibilidad de cada país. Este agente se une de manera irreversible al plasminógeno y bloquea su interacción con la fibrina. Su uso de forma local tiene una eficacia limitada para prevenir el sangrado oral ^38^^,^^39^.

#### Dolor posoperatorio

Los pacientes con enfermedad hepática poseen un metabolismo de fármacos impredecible. El metabolismo sufre una reducción marcada, lo que puede aumentar el efecto de los fármacos o causar una reacción inesperada. Se requiere realizar ajuste de dosis en aquellos medicamentos que tengan metabolismo hepático ^40^.

El paracetamol puede ser prescrito en dosis de 2 a 3 gramos al día en pacientes con hepatopatías. Es la recomendación de primera línea, incluso en pacientes con cirrosis alcohólica. Idealmente, no se debe superar la dosis de 2 gramos al día en este tipo de pacientes ^41^.

Los antiinflamatorios no esteroidales (AINE), a pesar de ser tolerados, deben ser evitados o utilizados con extrema precaución debido al riesgo de sangrado gastrointestinal. Este riesgo se ve agravado en pacientes con varices gastroesofágicas e hipertensión portal. Muchos AINE dependen del transporte de proteínas, por lo que los niveles pueden verse incrementados en pacientes con bajos niveles de proteína sérica; por lo tanto, si es necesario prescribirlos, debe hacerse en dosis más bajas de lo habitual ^40^^,^^42^.

El uso de opiáceos también debe ser prescrito con cautela debido al aumento de la toxicidad en casos de hipoalbuminemia asociada a cirrosis. Deben prescribirse en dosis bajas, solo si es necesario ^43^.

## CONCLUSIONES

Se debe valorar la atención odontológica del paciente con hepatopatías, según el motivo de consulta, el control de la enfermedad, la complejidad del procedimiento a realizar y medidas hemostáticas tanto intra como posoperatorias. A pesar de que se reporta una baja incidencia de eventos hemorrágicos posteriores a los procedimientos de cirugía oral menor, se deben considerar todas las medidas hemostáticas necesarias y considerar ajustes de dosis en el uso de AINE.

## Fuente de financiamiento:

Este trabajo no posee fuentes de financiamiento.
